# Non-volatile optoelectronic memory based on a photosensitive dielectric

**DOI:** 10.1038/s41467-023-40938-y

**Published:** 2023-09-05

**Authors:** Rui Zhu, Huili Liang, Shangfeng Liu, Ye Yuan, Xinqiang Wang, Francis Chi-Chung Ling, Andrej Kuznetsov, Guangyu Zhang, Zengxia Mei

**Affiliations:** 1 Songshan Lake Materials Laboratory, 523808 Dongguan, Guangdong P. R. China; 2 Institute of Physics, Chinese Academy of Sciences, 100190 Beijing, P. R. China; 3 State Key Laboratory for Mesoscopic Physics and Frontiers Science Center for Nano-optoelectronics, School of Physics, Peking University, 100871 Beijing, P. R. China; 4 Department of Physics, The University of Hong Kong, 999077 Hong Kong, P. R. China; 5 Department of Physics, University of Oslo, P.O. Box 1048, Oslo, NO-0316 Norway

**Keywords:** Photonic devices, Information storage, Optical data storage, Optoelectronic devices and components

## Abstract

Recently, the optoelectronic memory is capturing growing attention due to its integrated function of sense and memory as well as multilevel storage ability. Although tens of states have been reported in literature, there are still three obvious deficiencies in most of the optoelectronic memories: large programming voltage (>20 V), high optical power density (>1 mW cm^−2^), and poor compatibility originating from the over-reliance on channel materials. Here, we firstly propose an optoelectronic memory based on a new photosensitive dielectric (PSD) architecture. Data writing and erasing are realized by using an optical pulse to switch on the PSD. The unique design enables the memory to work with a programming voltage and optical power density as low as 4 V and 160 µW cm^−2^, respectively. Meanwhile, this device may be extended to different kinds of transistors for specific applications. Our discovery offers a brand-new direction for non-volatile optoelectronic memories with low energy consumption.

## Abstract (web summary)

Designing high efficient optoelectronic memory remains a challenge. Here, the authors report a novel optoelectronic memory device based on a photosensitive dielectric that is an insulator in dark and a semiconductor under irradiation with multilevel storage ability, low energy consumption and good compatibility.

## Introduction

**Fig. 1:**
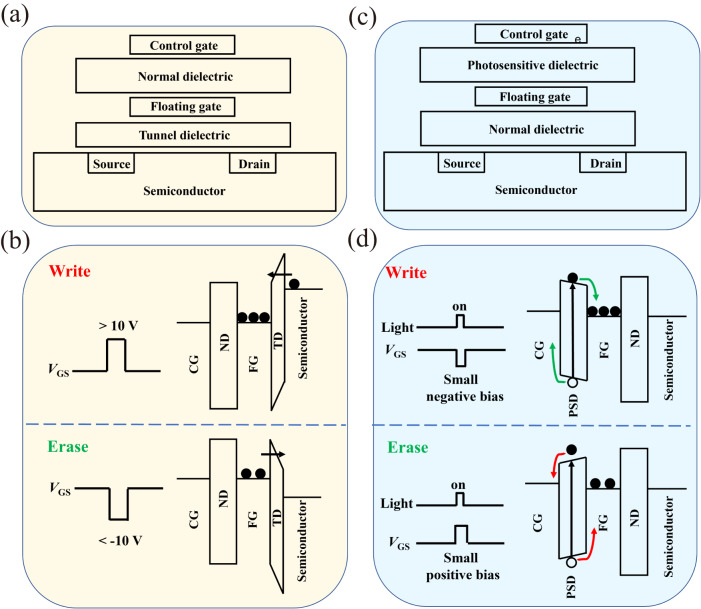
Difference between flash memory and PSD-based optoelectronic memory in terms of the device structure and write and erase processes. **a** Flash memory structure. **b** Write and erase processes in the flash memory. **c** Structure of the PSD-based optoelectronic memory. **d** Write and erase processes in the PSD-based optoelectronic memory. The solid balls represent the electrons, and the hollow balls represent the holes. The arrows represent flow directions of electrons or holes.

The emergence of smart home, autonomous driving, and bionic robots etc. marks the arrival of big data era, which puts forward increasingly higher requirements for nonvolatile memory, one of the carriers of information storage^1^. Flash memory has always been in an unshakable position in nonvolatile memory field^2–4^, although some new structures or principles, such as magnetic random-access memory^5^, ferroelectric random-access memory^6^, phase-change memory^7^ and resistive random-access memory^8^, were constantly proposed. However, faced with the already-arrived big data era, the drawbacks of flash memory have gradually emerged. Firstly, general flash memory cell has a two-state storage capacity, and the integration density is being restricted by the scaling down limit^9^, which creates a huge gap between the considerable data generated every day and the existing storage capability. Secondly, the programming voltage of flash memory has been maintained above 10 V, leading to a huge energy consumption^4^. The reason lies in its core structure and programming process, as shown in Fig. 1a, b. The difference between the flash memory and the common metal–oxide–semiconductor field-effect transistor (MOSFET) is the insertion of a 9–10 nm tunneling dielectric (TD) layer and a floating gate (FG) surrounded by the dielectric layers. Writing or erasing data depends on the electrons’ transport through the TD layer back and forth via Fowler–Northeim tunneling^10^ process and the resulting shift of the threshold voltage, which inevitably requires a large gate voltage (*V*_GS_) pulse (Fig. 1b).

The property of electrical read-out orthogonal to the photo-programming operation promises high differentiation among different data levels and imparts the ability of multilevel storage to optoelectronic memory^9^. For instance, different electrical signals, namely multi-states, can be obtained by adjusting the wavelength, the pulse width, the pulse number, the optical power density and even the polarization of the optical pulse.

Currently, three storage mechanisms were mainly adopted in optoelectronic memories: photochromic effect^10^, defects trapping of photogenerated carriers^11–17^, and tunneling of photogenerated carriers^1,18,19^. For photochromic memories, high optical power density is essential to break or connect the bonds in the polymer materials and modulate their resistance. A high-programming voltage is then a major requisite for the electrical regulation of the polymers. In the case of defects trapping in channel or interface, the photogenerated carriers will be captured in the defects and released slowly after turning off the optical pulse, known as persistent photoconductivity (PPC) effect. Because only a fraction of photogenerated carriers can be trapped by defects, the high optical intensity is needed to provide enough photogenerated carriers. Meanwhile, a large *V*_GS_ needs to be exerted to release the carriers trapped by the defects. The third-type memory device utilizes the tunneling of the photogenerated carriers in channel through the TD to FG under the large *V*_GS_. Similarly, because the probability of tunneling is small, the high optical power density is needed to provide enough photogenerated carriers into the channel.

As discussed above, a situation of high energy consumption will be inevitably encountered, considering the fact that the programming voltage and optical power density used in the previously reported optoelectronic memories were in excess of 20 V and 1 mW m^−2^, respectively. In addition, the strong dependence of these structures on the selection of channel materials and the control of defects makes it difficult to adapt to certain situations with different channel materials. It is urgently needed to develop new storage structure and working principle to realize the optoelectronic memory with low energy consumption and improved compatibility.

## Results

### Design of novel optoelectronic memory based on the PSD

A new optoelectronic memory structure with a photosensitive dielectric (PSD) is designed on the basis of the flash memory cell. As shown in Fig. 1c, the original normal dielectric (ND) layer in the flash memory (Fig. 1a) is replaced with a PSD layer, and the TD layer between the FG and the channel is replaced with an ND layer. Critically, the dielectric layer instead of the channel serves as the photosensitive component. The new PSD should own three significant features—(i) it works as a dielectric layer in dark, that is, it is insulating; (ii) it acts as a semiconductor and produce photogenerated carriers under illumination; (iii) it forms a distinct band offset with the FG layer and offers an energy barrier sufficient to block the carriers trapped in the FG.

It can be recognized that, with the assistance of an optical excitation with a suitable wavelength matching the bandgap of the PSD layer, the injection of electrons will be achieved by applying a small negative *V*_GS_ pulse, as indicated by the green arrows in Fig. 1d. Under the illumination, the photogenerated electrons in the PSD layer will drift to the FG layer driven by the negative *V*_GS_. After turning off the optical pulse, the PSD immediately becomes insulating, and the electrons in the FG are bound in the potential well formed by the energy barriers between the PSD, FG, and ND separately. In this case, the threshold voltage (*V*_th_) of the transistor will positively shift^2^, therefore accomplishing the writing and storage process of “0” state.

Correspondingly, extraction of electrons will be achieved by applying a small positive *V*_GS_ pulse combined with a similar optical pulse, as indicated by the red arrows in Fig. 1d. The electrons bounded in the FG will easily transport across the original barrier between the FG and PSD due to illumination-induced conductivity change in the PSD layer. Once stopping the optical pulse, the PSD immediately becomes insulating again, no electrons stay in the FG, and the threshold voltage will shift to the negative direction, erasing the “0” state and storaging “1” state.

In contrast to the optoelectronic memory reported in literature, the PSD-based memory introduces an innovative approach of injecting and extracting carriers with low power consumption as well as avoiding tradeoffs with choosing specific channel materials. The first and foremost, since the programming voltage is only used to guide the transport direction of the photogenerated carriers originated from the PSD, its value does not need to be large. Another outstanding merit of the new architecture is the efficient use of the intrinsic excitation-generated carriers in PSD for charge storage. It will remarkably decrease the need for a high optical power density. Thirdly, photogenerated carriers are generated in the PSD layer rather than in the general channel layer. It indicates that this new idea may feasibly work in different types of transistors, with less selectivity for channel materials (e.g., Si, oxides, 2D materials).

### The fabrication and performance of the novel PSD-based optoelectronic memory

The key for realizing the designed structure and mechanism is to find a suitable PSD material that exhibits the above-mentioned three features. Recently, Ga_2_O_3_ ultra-wide bandgap semiconductor (∼5.0 eV^20^) has attracted considerable attention in the solar-blind UV detection research field^20–23^. Zheng et al.^24^ and Kaya et al.^25^ reported the use of Ga_2_O_3_ a the high-K dielectric layer for MOSFETs in 2007 and 2016, respectively. It should be noticed that the electron affinity of Ga_2_O_3_ is ~4 eV, which enables the formation of a sufficiently high band offset between Ga_2_O_3_ and metals with high work function, such as Au having the work function of 5.2 eV^26^. These data suggest that Ga_2_O_3_ may satisfy all tradeoffs as the PSD we are looking for.

**Fig. 2:**
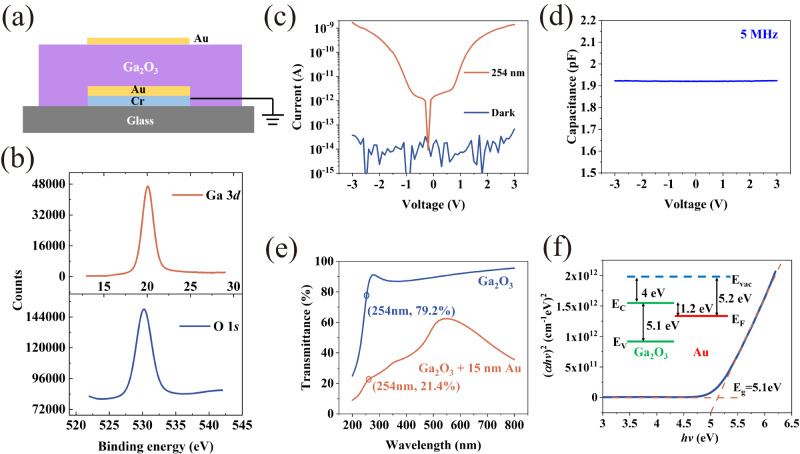
Characterization of the vertical structure based on Ga_2_O_3_ film. **a** Schematic diagram of the vertical structure. **b** XPS spectra of the Ga_2_O_3_ film. The upper and lower panels show the core level spectra of Ga 3*d* and O 1 *s*. **c** I–V curves in dark (blue curve) and under 254 nm illumination (orange curve). **d** C–V sweep curve at 5 MHz. **e** Optical transmittance spectra of the Ga_2_O_3_ (45 nm) film (blue curve) and the Ga_2_O_3_ (45 nm) + Au (15 nm) film (orange curve). **f** The plot of (α*h*ν)^2^ versus *h*ν for Ga_2_O_3_ film. The inset is the energy band structure of Ga_2_O_3_ and Au.

To start with, we prepared a two-terminal vertical structure based on Ga_2_O_3_, which is the top and core part of the PSD-based optoelectronic memory. The schematic diagram and top view of the magnified micrograph of the fabricated device are shown in Fig. 2a and Supplementary Fig. S1, respectively. Note that no diffraction peaks were observed in the XRD curve of the Ga_2_O_3_ film (Supplementary Fig. S2), proving the amorphous nature of the Ga_2_O_3_ deposited by radio-frequency magnetron sputtering at room temperature. Figure 2b demonstrates the core level spectra of O 1 *s* and Ga 3*d* measured by X-ray photoelectron spectroscopy (XPS), and Ga:O atomic ratio approaches 2:3 determined by the ratio of the integrated photoelectron peak area of O 1 *s* to Ga 3*d*. It suggests fewer oxygen vacancy defects in the Ga_2_O_3_ film and thus lower dark current in the device^23^. As shown in Fig. 2c, the dark current of the vertical structure is maintained at the ∼10^−14 ^A level, which is basically the limit of the source meter. In addition, a C–V test is conducted on the vertical structure at 5 MHz (Fig. 2d) and a relative permittivity value is extracted as 13.01, which shows that Ga_2_O_3_ can work as an insulating dielectric layer. The transmission spectrum of the Ga_2_O_3_ film is illustrated in Fig. 2e, with a direct bandgap of ~5.1 eV by Tauc’s law, as shown in Fig. 2f. It means that the Ga_2_O_3_ film has strong absorption for light whose wavelength locates at around 250 nm. Correspondingly, an apparent photoresponse is observed in Ga_2_O_3_ under 254 nm irradiation (Fig. 2c), satisfying the second requirement of the PSD as a semiconductor under suitable illumination. In addition, to decrease the absorption of control gate (CG) in the final device (Fig. 1c), 15 nm half-transparent Au was deposited on Ga_2_O_3_ as CG. The result manifests that about 50% of light is still absorbed and reflected by the top CG (Fig. 2e).

For the ultimate demonstration, we constructed a top-gate InGaZnO (IGZO) thin film transistor (TFT), the bottom part of the PSD-based optoelectronic memory. The schematic diagram and the top view magnified micrograph of the fabricated device are shown in Supplementary Fig. S3a,S3b, respectively. The initial linear region of the output characteristic curve (Supplementary Fig. S3c) clearly indicates the formation of a good ohmic contact between the source/drain electrodes and the channel. The transfer characteristic curve (Supplementary Fig. S3d) shows that the device has normal TFT performance with a high on-off ratio (>10^8^), which is the precondition for a large memory window.

**Fig. 3:**
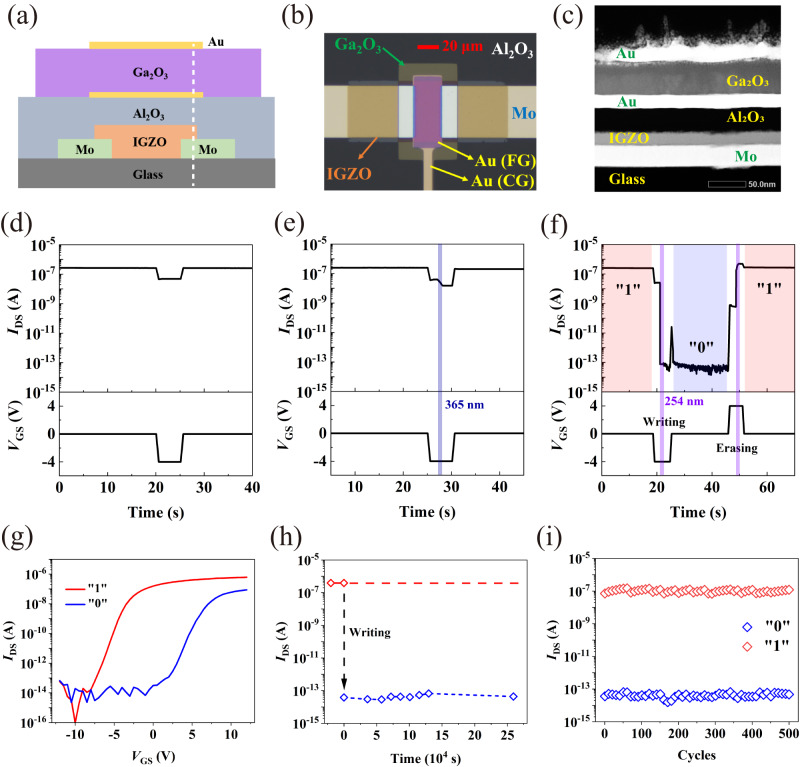
The structure and performance of the final PSD-based optoelectronic memory device. **a** Schematic diagram of the device. **b** Top view of the magnified optical micrograph of the fabricated device. **c** TEM cross-sectional image corresponding to the position of the white dashed line in (**a**). **d** The writing result by *V*_GS_ = −4 V pulse. **e** The writing result by a combination of *V*_GS_ = −4 V pulse and a 365 nm of 160 µW cm^−2^ optical pulse. **f** The single write/erase process and corresponding results. (optical pulse: 254 nm, 160 µW cm^−2^, 1 s; *V*_GS_ pulse: −4V / 4 V). **g** Transfer characteristic curves in states “0” and “1”. **h** Retention test for up to 72 h. (optical pulse: 254 nm, 160 µW cm^−2^, 1 s; *V*_GS_ pulse: −4 V). **i** Fatigue test result for 500 cycles. (optical pulse: 254 nm, 160 µW cm^−2^, 1 s; *V*_GS_ pulse: −4 V/4 V). **g**–**i** Blue represents “0”, and red represents “1”.

The two-terminal Ga_2_O_3_ vertical structure and top-gate IGZO TFT are stacked together to compose the final PSD-based optoelectronic memory device. Figure 3a, b show the schematic diagram and top view of the magnified micrograph of the final device, respectively. Figure 3c is the cross-sectional TEM image, where Au, Ga_2_O_3_, Au, Al_2_O_3_, IGZO, and Mo layers are clearly observed exhibiting sharp interfaces. To avoid the possible contaminations of the photoresist at the interfaces, each lithography process is optimized as shown in Supplementary Fig. S4. Importantly, the band alignment of this memory structure matches the new conception introduced in Fig. 1d, as confirmed in Supplementary Fig. S5.

The transfer characteristics of the PSD-based optoelectronic memory (Supplementary Fig. S6) basically retain the performance of the isolated IGZO TFT, except slightly larger subthreshold swing (*SS*) value and more negative threshold voltage (*V*_th_). That is ascribed to a smaller capacitance caused by the series connection of the two capacitors (CG/PSD/FG + FG/ND/channel) in the device.

It is clearly demonstrated that only applying negative *V*_GS_ pulses is not sufficient to inject charges into the FG, as shown in Fig. 3d, as there is no light to turn on the PSD. Moreover a combination of the negative *V*_GS_ pulse with a 365 nm optical pulse cannot cause the carriers’ injection either (Fig. 3e), since 365 nm does not match the bandgap of Ga_2_O_3_.

The single writing and erasing process and their results are shown in Fig. 3f to verify the memory function of the final device. *V*_GS_ and the drain voltage (*V*_DS_) under read status were 0 V and −0.5 V, respectively. In the initial state “1”, the drain current (*I*_DS_) is stably maintained at ~3 × 10^−7 ^A. After applying a *V*_GS_ pulse = −4 V and a 254 nm optical pulse with as low as 160 µW cm^−2^ power, *I*_DS_ is stably maintained at ~5 × 10^−14 ^A, corresponding to “0” state with a memory window >10^6^. As expected, the electrons are injected into the FG from the CG and trapped in the FG in this process, causing a right shift of the threshold voltage **(**Fig. 3g). Then we apply a *V*_GS_ = 4 V pulse and an identical optical pulse, and the *I*_DS_ recovered to the initial value, ~3 × 10^−7 ^A. The phenomenon indicates that after the 254 nm light “turns on” Ga_2_O_3_, the electrons stored in the FG are rapidly released back to the CG under the electric field induced by the small positive *V*_GS_ and net electrons in FG. Apparently, the electrons’ transport direction can be discerned from the synchronous variation of the gate current (*I*_GS_) during the writing and erasing operations of *I*_DS_, as shown in Supplementary Fig. S7. During the writing process, the negative *I*_GS_ (process “B”) proves that electrons flow from CG to FG, while the positive *I*_GS_ (process “C”) proves that electrons flow from FG to CG during the erasing process. The in-situ monitoring results of the electrons’ transport behavior perfectly is in accordance with the designed injection/extraction processes highlighted by green and red arrows in Fig. 1d, solidly corroborating the feasibility of the working principle in PSD-based optoelectronic memory.

The data retention time is an important parameter of the device reliability. As shown in Fig. 3h, the memory window constantly keeps >10^6^ even after 72 h. The original data of retention test are shown in Supplementary Fig. S8. Fatigue characteristic is another important parameter of the device reliability. The cyclic program/erase (P/E) endurance of the device was examined and exhibited in Fig. 3i. After 500 cycles, the memory window has almost not changed.

**Fig. 4:**
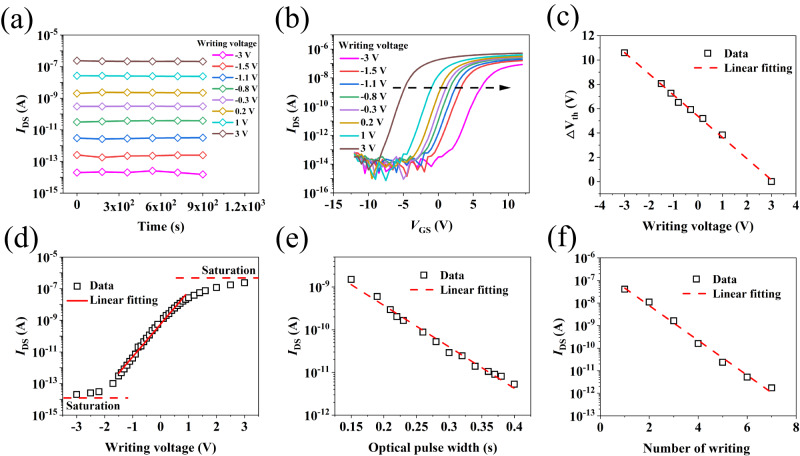
Tests of the multilevel storage. **a** Retention test of different states formed by different *V*_GS_ pulses (keeping the optical pulse at 254 nm, 160 µW cm^−2^, 1 s). The corresponding relationship between color and writing voltage is shown in the legend. **b** Transfer curves of different states formed by different *V*_GS_ pulses. The corresponding relationship between color and writing voltage is shown in the left legends. **c** Linear fitting of ∆*V*th for different states formed by different *V*_GS_ pulse. The *V*_th_ is defined as the value of *V*_GS_ when *I*_DS_ = (*W*/*L*) × 1 nA, *W* and *L* are width and length of the channel, respectively. **d** Linear fitting of *I*_DS_ for different states formed by different *V*_GS_ pulse. **e** Linear fitting of *I*_DS_ for different states formed by different optical pulse width (optical pulse: 254 nm, 160 µW cm^−2^; *V*_GS_ pulse: −4 V). **f** Linear fitting of *I*_DS_ for different states formed by different number of writing (optical pulse: 254 nm, 60 µW cm^−2^, 50 ms; *V*_GS_ pulse: −3 V).

Compared with conventional three-terminal transistor devices, the addition of optical pulse provides a new route to multilevel storage in nonvolatile memories. Here, three different methods are explored to illustrate the multilevel storage capability. First, we change the amplitude of the *V*_GS_ pulse to implement multilevel storage. As shown in Fig. 4a, the more negative *V*_GS_ is, the more electrons are injected into the FG, and the lower *I*_DS_ is. During the 15 min retention test, every state keeps stable. Figure 4b shows the transfer curves corresponding to every state. The more negative the *V*_GS_ is, the more the curve right shifts. Figure 4c shows that *V*_th_ shifts (∆*V*_th_) extracted from Fig. 4b have a good linear relationship with the amplitude of *V*_GS_ pulse. Linear fitting result of *I*_DS_ for different states indicates in a write voltage between −2 V and 1 V, corresponding to a current storage window range of approximately 6 orders of magnitude, we can obtain a large number of linearly related storage states, as shown in Fig. 4d. Then, by changing the optical pulse width and the number of writings, we can also obtain a linearly related multilevel storage states, as shown in Fig. 4e, f.

Table 1 compares key parameters of the PSD-based optoelectronic memory proposed by us with previously reported ones. The new memory exhibits comparable performance regarding memory window, retention time, and fatigue cycles. The programming voltage and optical power density are particularly far lower than those ever-reported values, which reveals the contribution of PSD in the novel structure.

**Table 1:** Comparison of key parameters with previously reported optoelectronic memories

Working principle	Photosensitive material	Programming voltage	OPD [mW/cm^2^]	Programming time	Memory window	Tested retention time	Tested fatigue cycles	Ref.
Photochromic	DAEs	60 V	–	5 s	>10^5^	500 days	70	^10^
Tunneling	MoS_2_	100 V	5 × 10^4^	1 s	>10^7^	10^4^ s	70	^18^
Tunneling	SnS_2_	40 V	–	5 s	>10^4^	–	–	^1^
Tunneling	PtS_2_	30 V	41	100 ms	>10^3^	–	–	^19^
Defects	BTBT	120 V	40	12 s	>10^6^	2 × 10^4^ s	–	^11^
Defects	MoS_2_/PbS	40 V	0.027	5 s	<3	10^4^ s	2000	^12^
Defects	WSe_2_	80 V	210	5 s	10^6^	4.5 × 10^4^ s	200	^13^
Defects	Penance	60 V	71.59	120 s	10^4^	100 days	3	^14^
Defects	PDVT-10:N2200	40 V	5	1 s	10^5^	2 × 10^4^ s	500	^15^
Defects	MoS_2_	80 V	501	10 s	10^7^	10^4^ s	250	^16^
Defects	TiO_2_	50 V	1	30 s	<2	10^4^ s	–	^17^
PSD	Ga_2_O_3_	4 V	0.16	1 s	>10^6^	2.6 × 10^5^ s	500	This work

*OPD* optical power density.

To get closer to the practical application, we use an AlGaN UV-LED^27^ as the light source to realize the programming function of the PSD-based optoelectronic memory. The electroluminescence spectrum of the UV-LED is shown in Supplementary Fig. S9a. The photograph of the test configuration is exhibited in Supplementary Fig. S9b. As seen from these data, the function of writing and erasing has been successfully achieved by using the combination of AlGaN UV-LED and Ga_2_O_3_ PSD-based optoelectronic memory (Supplementary Fig. S9c). This demonstration paves the way for future integration of optoelectronic memory circuits.

## Discussion

The optoelectronic memory with IGZO as a channel and Ga_2_O_3_ as the PSD is the first example of PSD-based device architecture not only conceptually proposed, but also tested in experimental conditions. It paves the way to explore more PSD candidates and various channel materials. In addition, the memory performance can be further optimized. For instance, longer retention and faster writing speed may be potentially achieved by reducing the defect in PSD. By optimizing the *SS* parameter of the transistor, a larger memory window can be obtained under the same write conditions, or a smaller programming voltage and optical power density be obtained under the same memory window. The number of multilevel storages can also increase with more subdivided write methods or larger memory windows.

## Results and discussion

In summary, with the booming development of ultra-wide-bandgap semiconductors, the boundary between semiconductors and insulators has become more and more blurred. Here, Ga_2_O_3_ is opted as the PSD material in novel nonvolatile optoelectronic memory, considering its admirable insulating property in dark and semiconducting performance under illumination. Via integrating the Ga_2_O_3_ PSD with the common floating gate device, unique programming methods are established with low programming voltage (4 V), low optical power density (160 µW cm^−2^), and multilevel storage (storage window of approximately six orders of magnitude with good linear relationship). This memory may potentially fill the gap between big data and limited storage capacity as well as reducing the energy consumption. Meanwhile, the new structure and mechanism can be readily extended to other FETs compatible with photolithography, such as Si-based, organic or 2D materials-based FETs, to achieve high-performance solid-state memory and flexible memory. Remarkably, the device can also be used as a multi-function sensor, integrating the sensing and storage abilities together^28,29^.

## Methods

### Preparation of a two-terminal vertical structure based on Ga_2_O_3_

First, the glass substrate is sonicated with acetone, alcohol, and deionized water for 10 min in sequence. After drying the glass substrate with nitrogen, 10 nm Cr layer is deposited by radio-frequency (RF) magnetron sputtering to improve the adhesion of Au (32 nm) on glass, followed by using photolithography and etching into bottom electrode pattern. Then Ga_2_O_3_ (45 nm) is deposited by RF magnetron sputtering (60 W) in an oxygen-rich condition, and 15 nm Au is then sputtered on Ga_2_O_3_. Patterning of the top electrode and gallium oxide is completed in sequence. Finally, the device is annealed at 200 °C for 30 min in air.

### Preparation of IGZO top-gate TFT

First, use the same method to clean the glass substrate. Then grow Mo (30 nm) by magnetron sputtering, and define the channel length (20 µm) and width (50 µm) by photolithography. Then grow IGZO (20 nm) by RF magnetron sputtering. After IGZO is patterned, Al_2_O_3_ (30 nm) is grown by atomic layer deposition (ALD) at 200 °C. Then Au (15 nm) is deposited by RF magnetron sputtering. After that, Au is etched to form the gate pattern, and the aluminum oxide is etched to expose the source and drain electrodes for testing. Similarly, the device is annealed at 200 °C for 30 min in air.

### Preparation of PSD-based optoelectronic memory device

Based on the TFT manufacturing process, the original Au (10 nm) is etched to form the FG pattern. Then follow the same method to grow 45 nm Ga_2_O_3_ as the PSD layer and 15-nm semitransparent Au as the CG. Finally, Au, Ga_2_O_3_ and Al_2_O_3_ are etched in sequence. Similarly, the device is annealed at 200 °C for 30 min in air.

### Material characterization

The XPS measurements were conducted using a multifunctional X-ray Photoelectron Spectroscopy analysis platform (ESCALAB Xi + , ThermoFisher), where an electron source was used for charge compensation. TEM cross-sectional image was obtained by TEM (JEM-F200). The transmission spectrum was measured by UV–Vis–NIR Spectrophotometer (UH4150, Hatachi). The XRD curve was measured by an X-ray diffractometer (D8-ADVANCE, Bruker).

### Device characterization

Except for C–V test performed by Keysight B1500A and current source of AlGaN UV-LED provided by Keithley 2400, all other electrical tests were conducted by Keithley 2636B. The light source other than the LED is generated by an UV-enhanced xenon lamp, and the wavelength is adjusted by a monochromator. The generation of optical pulses is achieved by placing an electric switch between the xenon lamp and the monochromator. The optical power density is calibrated by a standard Si photodetector.

### Supplementary information

Supplementary Information

Peer Review File

## Data Availability

All raw data are available from the author upon request. Almost all of testing data shown in this manuscript are themselves raw I–V or I–t data instead of being processed.
