# Supplementary material for: Non-volatile optoelectronic memory based on a photosensitive dielectric
Source: Nat Commun. 2023 Sep 5;14:5396. doi: 10.1038/s41467-023-40938-y (PMC10480167; doi:10.1038/s41467-023-40938-y)
Supplement: Supplementary file 1 — Supplementary Information [file 41467_2023_40938_MOESM1_ESM.pdf]

A non-volatile optoelectronic memory based on photosensitive dielectric

Rui Zhu[1,2], Huili Liang[1,2], Shangfeng Liu[1,3], Ye Yuan[1], Xinqiang Wang[1,3], Francis Chi-Chung Ling[4], Andrej Kuznetsov[5], Guangyu Zhang[1,2], Zengxia Mei[1,2]**

[1] Songshan Lake Materials Laboratory, Dongguan, Guangdong 523808

[2] Institute of Physics, Chinese Academy of Sciences, Beijing 100190

[3] State Key Laboratory for Mesoscopic Physics and Frontiers Science Center for Nano-optoelectronics, School of Physics, Peking University, Beijing 100871

[4] Department of Physics, The University of Hong Kong, Hong Kong 999077, P. R. China

[5] Department of Physics, University of Oslo, Oslo P.O. Box 1048, NO-0316, Norway

Email: zxmei@iphy.ac.cn; zhurui@iphy.ac.cn

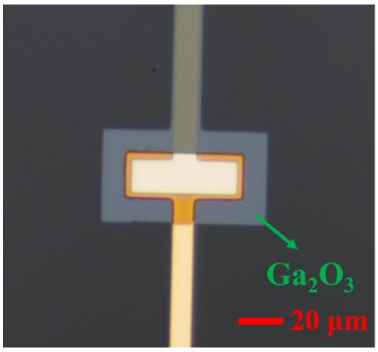

Figure S1. Top view of the magnified micrograph of the vertical structure based on Ga_2O_3.

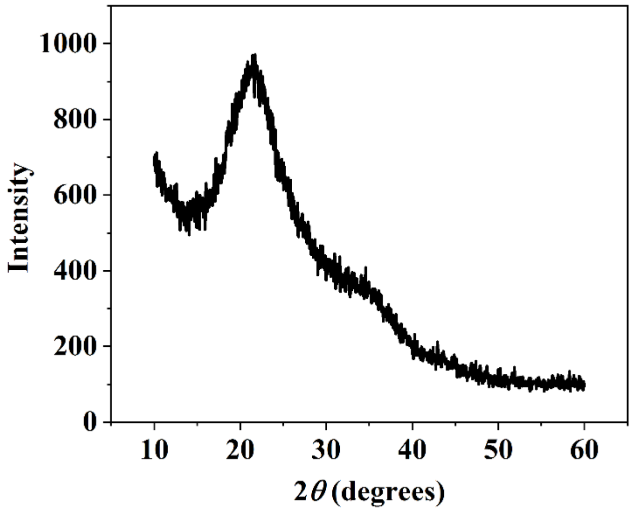

Figure S2. XRD curve of the Ga_2O_3 film on quartz.

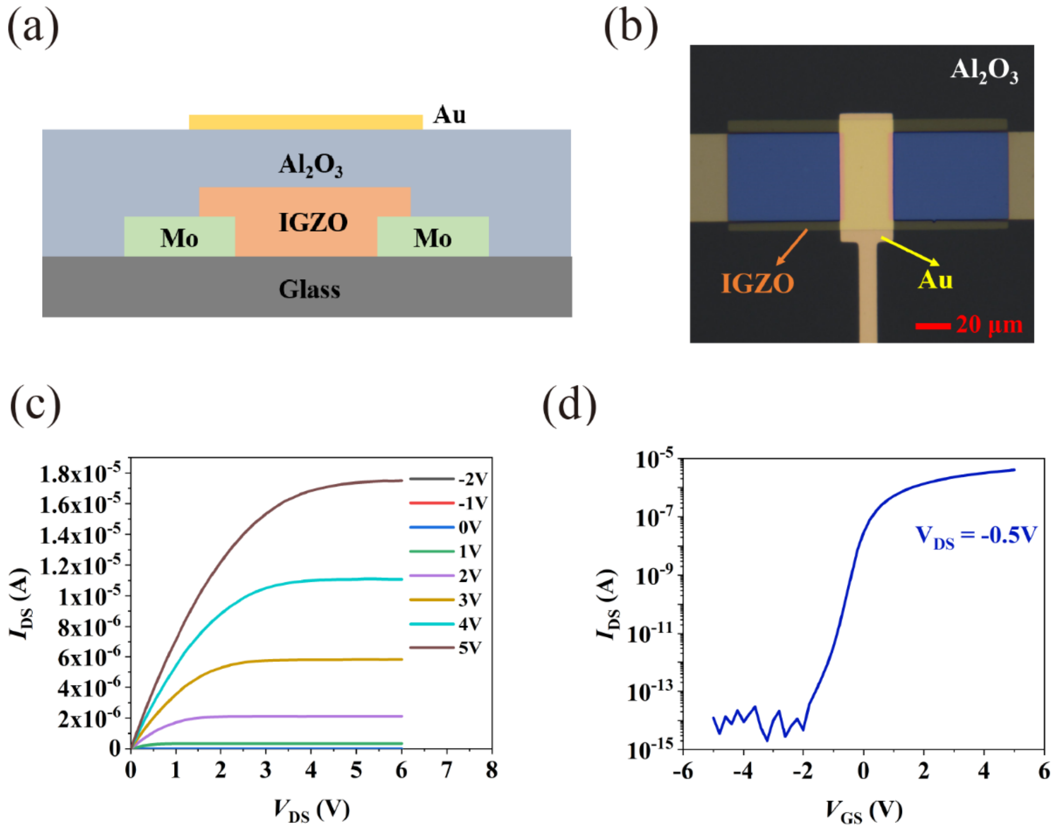

Figure S3. Fabrication and characterization of IGZO TFT. a) Schematic diagram of IGZO TFT. b) Top view of the magnified micrograph of fabricated device. c) Output curves. d) Transfer curve.

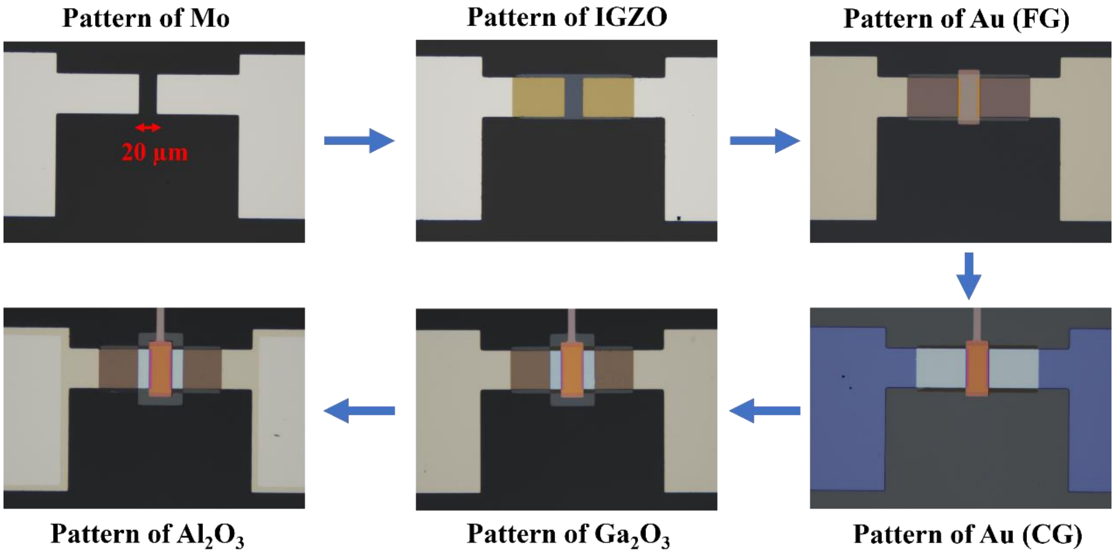

Figure S4. Fabrication process of PSD-based optoelectronic memory and top view of the magnified micrograph in each step.

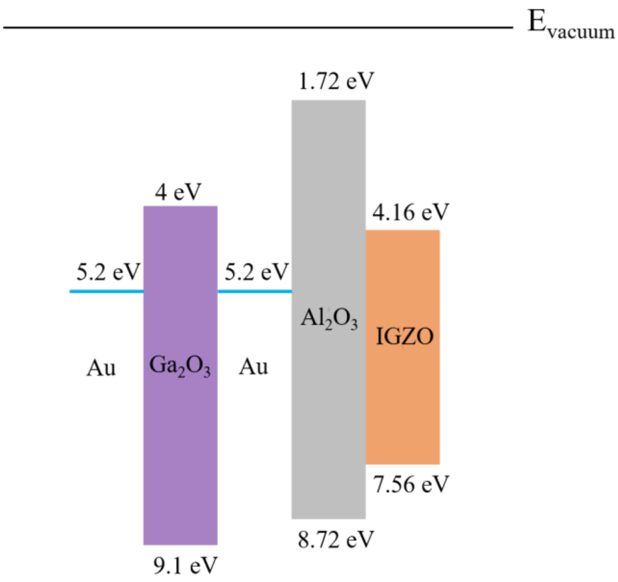

Figure S5. Band alignment diagram of the PSD-based memory device.

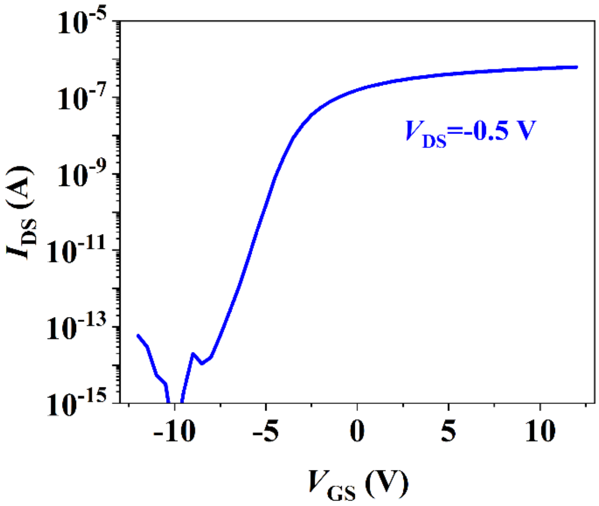

Figure S6. Transfer curve of PSD-based optoelectronic memory.

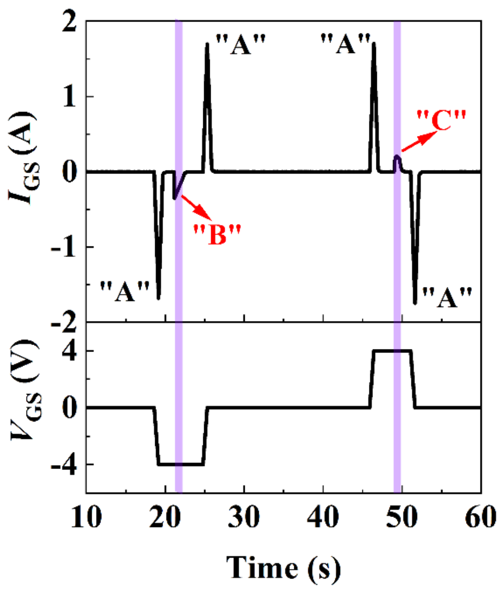

Figure S7. In-situ monitoring of gate current corresponding to the write and erase process in Figure 3f. Process ‘A’ represents the charge and discharge currents of the capacitor when the voltage suddenly changes. Process ‘B’ represents electrons injection into FG from CG. Process ‘C’ represents electrons extraction from FG to CG.

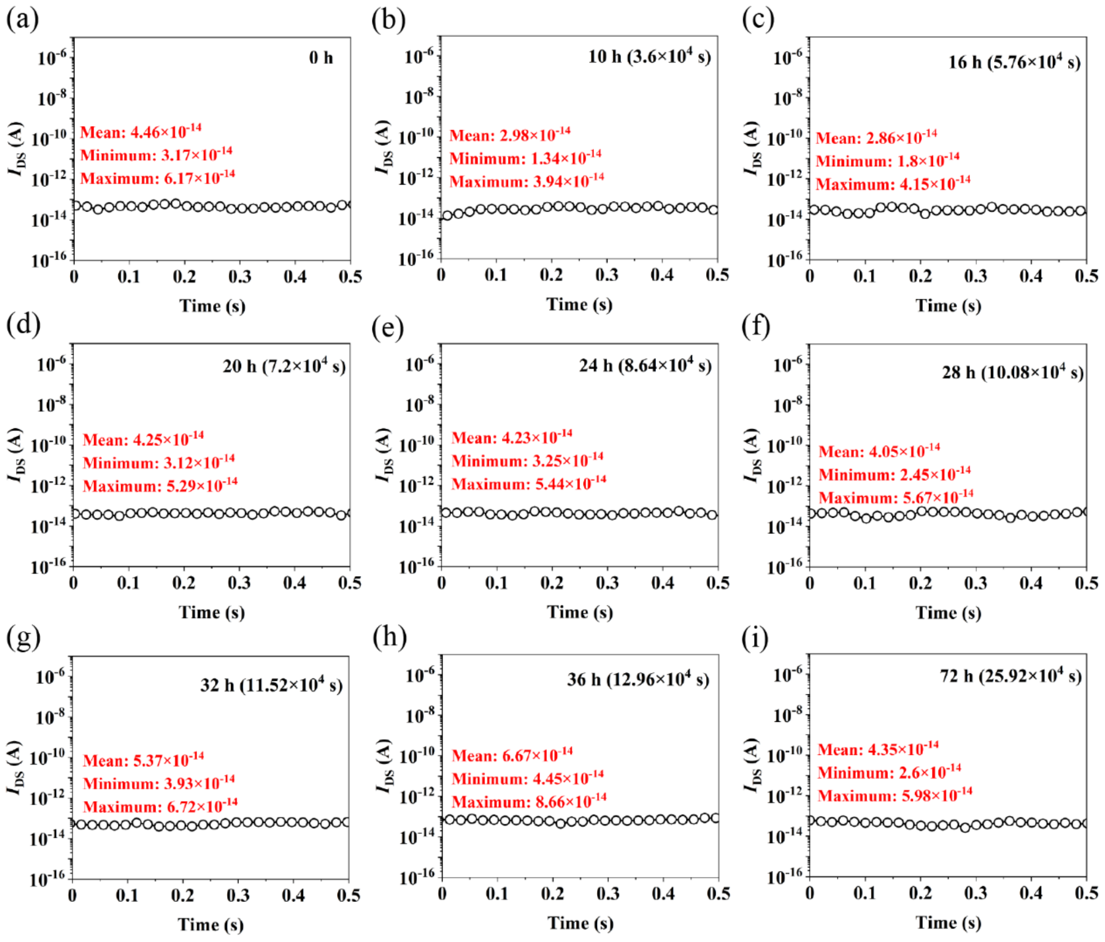

Figure S8. Original data of retention test. a) ~ i) Reading result after writing for 0, 10, 16, 20, 24, 28, 32, 36 and 72h. Each reading lasts for 0.5 s. Mean value of each reading result is used to form Figure 3h.

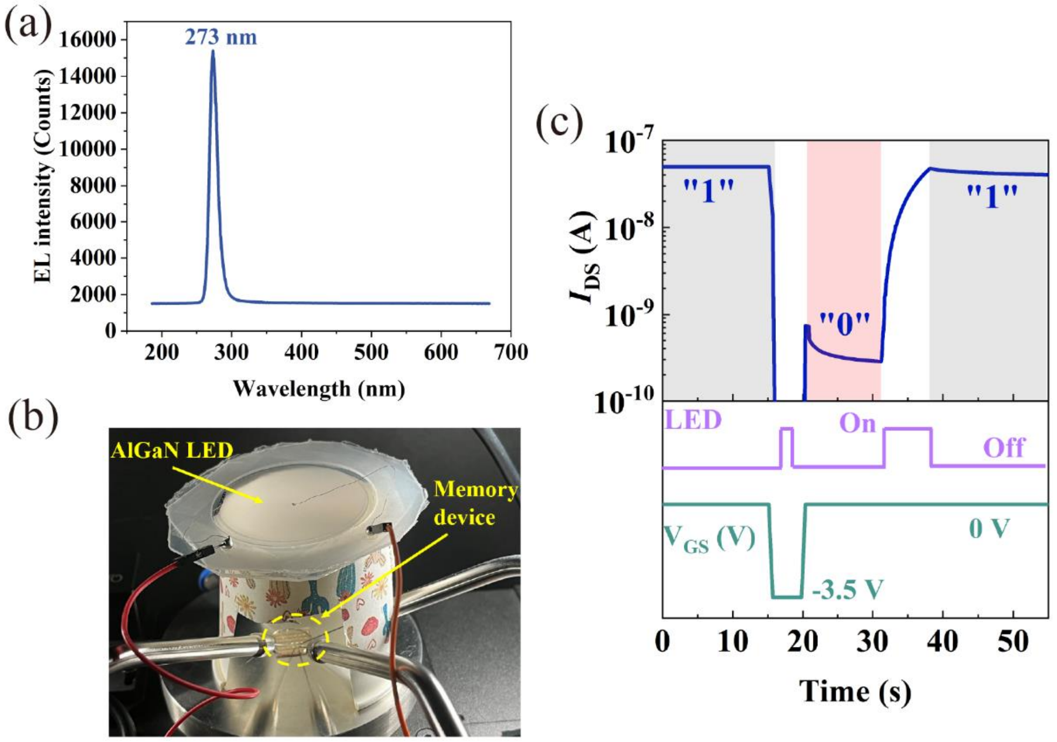

Figure S9. Memory test with AlGaN UV-LED as the light source. a) Electroluminescence spectrum of the UV-LED under 40 mA. b) Photograph of the simple test configuration. The AlGaN LED is located above the PSD-based optoelectronic memory. c) The single write and erase process and corresponding results. (V_{DS}: -0.5 V).
